# Coal-Free Zone Genesis and Logging Response Characterization Using a Multi-Curve Signal Analysis Framework

**DOI:** 10.3390/e27121183

**Published:** 2025-11-21

**Authors:** Xiao Yang, Yanrong Chen, Longqing Shi, Xingyue Qu, Song Fu

**Affiliations:** 1 College of Earth Sciences and Engineering, Shandong University of Science and Technology, Qingdao 266590, China; 202281030006@sdust.edu.cn (X.Y.); skd994985@sdust.edu.cn (X.Q.); 202181030005@sdust.edu.cn (S.F.); 2 School of Geosciences, China University of Petroleum (East China), Qingdao 266580, China; chenyanrong272371@163.com

**Keywords:** coal-free zone, coal seam scouring zone, logging signals, singular spectrum analysis (SSA), trend–fluctuation composite curve (TFC), water inrush risk evaluation

## Abstract

Coal-free zones, particularly scouring zones, reduce recoverable reserves and increase water inrush risk in coal mining. Existing sedimentological, geophysical, and geostatistical methods are often constrained by coring conditions, lithological interpretation accuracy, and geological complexity. Given that well log signals provide the most continuous carriers of geological information, this study integrates Singular Spectrum Analysis (SSA), Maximum Entropy Spectral Analysis (MESA), and Integrated Prediction Error Filter Analysis (INPEFA) to establish a multi-curve framework for analyzing the genesis and logging responses of coal-free zones. A two-stage SSA workflow was applied for noise reduction, and a Trend–Fluctuation Composite (TFC) curve was constructed to enhance depositional rhythm detection. The minimum singular value order (*N*), naturally derived from SSA-decomposed INPEFA curves, emerged as a quantitative indicator of mine water inrush risk. The results indicate that coal-free zones resulted from inhibited peat-swamp development followed by fluvial scouring and are characterized by dense inflection points and frequent cyclic fluctuations in TFC curves, together with the absence of low anomalies in natural gamma-ray logs. By integrating multi-curve logs, core data, and in-mine three-dimensional direct-current resistivity surveys, the genetic mechanisms and boundaries of coal-free zones were effectively delineated. The proposed framework enhances logging-based stratigraphic interpretation and provides practical support for working face layout and mine water hazard prevention.

## 1. Introduction

With the continuous intensification of coal resource exploitation in China [1], many mining areas are gradually advancing into deeper and marginal zones [2,3]. The presence of coal-free zones within coalfields has increasingly become a critical factor restricting safe and efficient mining [4]. Coal-free zones, especially coal seam scouring zones, are common geological phenomena in coal-bearing strata that often lead to thinning or even absence of coal seams [5]. This not only reduces recoverable reserves and constrains working face layout but may also trigger mine water inrush and coal and gas outburst hazards [6,7,8,9]. Therefore, analyzing the genetic mechanisms of coal-free zones and establishing refined identification methods are of great engineering significance for the rational development of coal resources and the prevention of mine disasters.

Existing studies on the genesis and identification of coal-free zones have primarily relied on sedimentological, geophysical, and geostatistical approaches [10,11,12]. Sedimentological methods are mostly based on variations in coal seam thickness, sandbody distribution, and facies evolution and delineate coal-free zones in a qualitative or semi-quantitative manner. Geophysical methods employ electrical and seismic techniques to reveal structural features within coal-bearing strata, while geostatistical methods utilize variogram models to quantify the presence of coal-free zones through hole-effect analysis. With the advancement of research, integrated multi-method approaches [13,14] have gradually become an important direction. However, they are still constrained by coring conditions, lithological interpretation accuracy, and geological complexity and thus require continuous validation in subsequent mining practice [15]. In contrast, identifying the logging responses of coal-free zones directly from well logs, supplemented by core observations and small-scale geophysical surveys, provides a more effective means of revealing their genetic characteristics and supporting working face layout [16,17,18].

In recent years, well-logging data have become one of the most direct and continuous sources of subsurface geological information and are increasingly used to reveal the sedimentary architecture of coal-bearing strata [19,20]. In particular, when continuous core recovery is not available, the natural gamma (GR) log, which is highly sensitive to clay content and lithological changes, has been widely applied to sedimentary cycle analysis and stratigraphic correlation [21,22]. Developments in signal processing and spectral analysis have further enhanced the ability of logs to capture geological information. Singular Spectrum Analysis (SSA) can separate noise from the main components without altering the overall morphology of the curve, thereby improving GR signal quality [23,24]. Maximum Entropy Spectral Analysis (MESA) helps identify the dominant frequency bands associated with sedimentary cycles [25]. INPEFA characterizes long-term base-level changes and has been widely applied in isochronous stratigraphic correlation, Milankovitch-cycle research, and reservoir studies [26,27]. Moreover, the formation of coal-free zones is commonly associated with increased depositional energy, enhanced base-level fluctuations, and sand-body scouring. These geological processes typically produce identifiable variations in the structure of well-logging signals. Therefore, the integrated use of SSA, MESA, and INPEFA makes it possible to extract key indicators of sedimentary evolution directly from logging curves, reduces the dependence on lithological interpretation accuracy, and provides a more reliable technical basis for analyzing the genesis of coal-free zones.

Against this background, this study focuses on the Tianchen coalfield in the eastern North China Plate and conducts a systematic investigation of the genesis of coal-free zones and their logging responses. A two-stage SSA is applied to the logging data for noise reduction and principal component extraction, and MESA is used to identify dominant sedimentary cyclicity and enhance curve interpretability. On this basis, a Trend–Fluctuation Composite (TFC) curve is constructed by combining the trend and fluctuation components of the INPEFA curve, and the minimum singular value order *N* derived from INPEFA decomposition is introduced as a quantitative indicator of lithological variability relevant to water-inrush assessment. Finally, by integrating the multi-curve recognition framework with core observations and in-mine 3D DC resistivity data, the genesis of the coal-free zone is clarified, and its boundary is refined.

## 2. Study Area and Data Sources

### 2.1. Geological Settings

**Figure 1:**
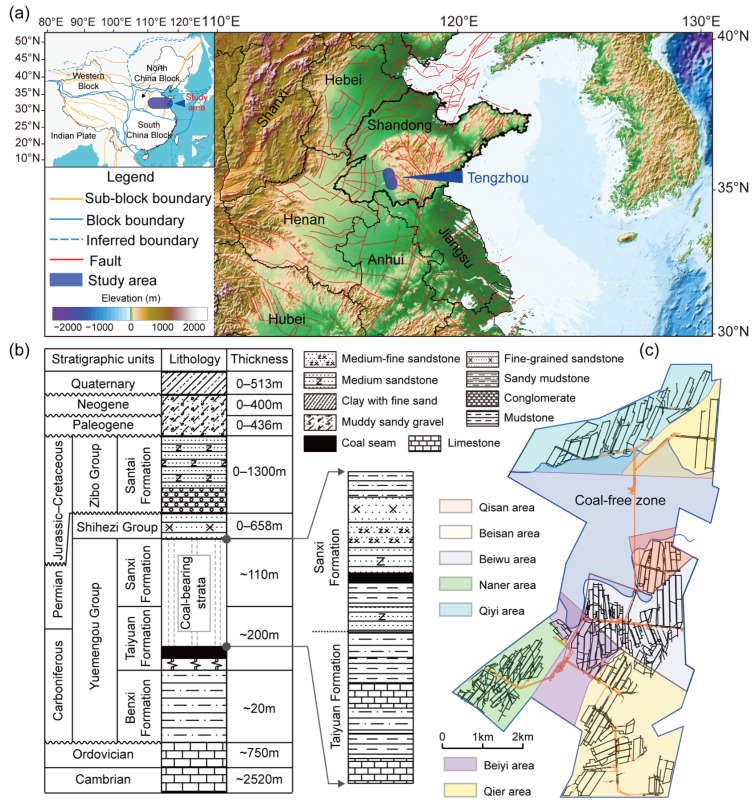
Study area overview: (**a**) Location; (**b**) Stratigraphic column; (**c**) Minefield layout.

The Tianchen coalfield is located in Tengzhou City, Shandong Province, in the eastern part of the North China Plate (Figure 1a). The main coal-bearing strata are the Carboniferous–Permian Shanxi and Taiyuan formations. The lower Shanxi Formation is dominated by grayish-white medium- to fine-grained sandstones interbedded with minor siltstone, sandy mudstone, and mudstone and serves as the primary host of the No. 3 coal seam. The Taiyuan Formation consists of dark gray mudstone, thin-bedded limestone, grayish-green sandstone, and minor claystone. The third limestone (L3) lies on average 46 m above the No. 3 coal seam of the Shanxi Formation and represents an important stratigraphic marker (Figure 1b). The No. 3 coal seam is characterized by stability, low sulfur content, shallow burial depth, and moderate coal rank. After 35 years of mining, recoverable resources remain mainly in the Qiyi and Qier areas in the northern part of the coalfield (Figure 1c).

### 2.2. Data Sources

**Figure 2:**
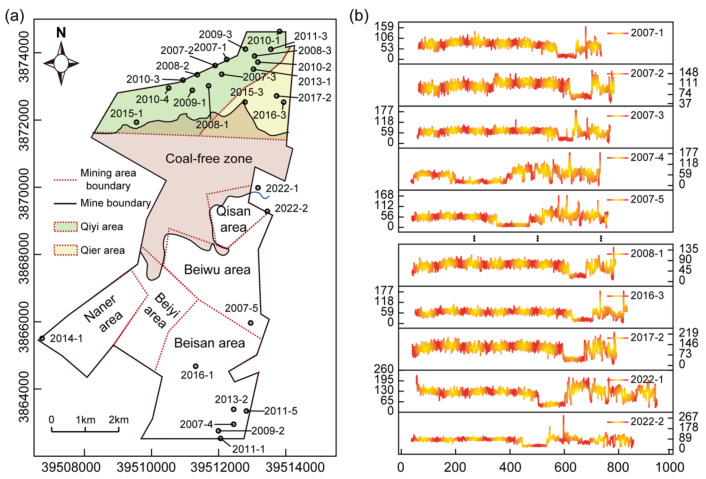
Natural gamma ray logging data: (**a**) Borehole distribution; (**b**) GR logging curves.

This study mainly used natural gamma ray (GR) logging data, collected from 28 boreholes in the Tianchen coalfield. GR logs are highly sensitive to clay content and lithological variations and can effectively reflect changes in depositional environments [28]. In fluvial depositional sequences, overlying fine-grained strata, mudstone, and coal seams commonly mark the transition from high- to low-energy conditions and the termination of a depositional cycle [29]. Therefore, GR logs provide a critical data basis for characterizing the logging responses of coal-free zones. The distribution of boreholes and representative GR logs are presented in Figure 2 and Table 1.

## 3. Methodology

This study was based on Natural Gamma (GR) logging data from 28 boreholes and employed two-stage Singular Spectrum Analysis (SSA), Maximum Entropy Spectral Analysis (MESA), and Integrated Prediction Error Filter Analysis (INPEFA) to systematically illustrate the workflow for characterizing the logging responses of coal-free zones. First, the GR logs were preprocessed and analyzed using the first stage of SSA to remove noise and extract the main components. Subsequently, MESA and INPEFA were applied to identify sedimentary cyclicity and long-term depositional trends in the logging data. Then, a second stage of SSA was conducted to further suppress noise in the INPEFA curves, and the minimum singular value order (*N*) was introduced as a quantitative indicator of lithological variability for evaluating aquifer richness and water inrush risk. Finally, three-dimensional high-density electrical surveys were integrated to delineate coal-free zone boundaries and verify their genetic mechanisms.

### 3.1. Maximum Entropy Spectral Analysis

Maximum Entropy Spectral Analysis (MESA) exhibits stratigraphic sensitivity in the vertical direction, where each cross-section represents the “local spectrum” at the current depth rather than a global average frequency. This enables the capture of frequency energy characteristics that vary with depth, thereby more precisely revealing the structure of sedimentary cycles [25]. MESA is a method for extrapolating the autocorrelation function based on the maximum entropy criterion. The basic principle is as follows:

For a stationary random process, the relationship between the power spectral density function *S_x_*(*ω*) and the autocorrelation function *r*(*m*) is defined by the Wiener–Khinchin theorem:

(1) $$S_{x}(\omega )=\sum_{m=-\infty }^{\infty }r_{x}(m)e^{-j\omega m}$$

If the values of the autocorrelation function *r*(*m*) are known for all *m*, the power spectral density function can be calculated using the above equation. However, for a finite-length sequence, only a limited number of autocorrelation values can be estimated (Equation (2)), and the remaining values are assumed to be zero (Equation (3)).

(2) $$S_{x}(\omega )=\sum_{m=-M}^{\infty }r_{x}(m)e^{-j\omega m}$$

(3) $$r_{x}(m)=0\left(\left|m\right|>M\right)$$

In Equation (3), *M* is the maximum lag used in calculating the autocorrelation function. To address truncation errors in spectral estimation, the method extrapolates the (*M* + 1)-th value of the autocorrelation function from the first *M* values of the known autocovariance function (*r*_1_, *r*_2_, ⋯, *r_M_*) while ensuring that the sequence always maintains maximum entropy (Equation (4)). The sequence entropy is given by Equation (5), and the covariance matrix is shown in Equation (6):

(4) $$\partial H(x)/\partial r_{M+1}=0$$

(5) $$H(x)=\frac{M+1}{2}\ln(2\pi e)+\frac{1}{2}\ln\left(\det R_{M+1}\right)$$

(6) $$R_{M+1}=\left[\begin{array}{cccc} r_{0} & r_{1} & \ldots & r_{M+1}\\ r_{1} & r_{0} & \ldots & r_{M}\\ \vdots & \vdots & \ddots & \vdots \\ r_{M} & r_{M-1} & \ldots & r_{1}\\ r_{M+1} & r_{M} & \ldots & r_{0} \end{array}\right]$$

Therefore, maximizing the entropy function (Equation (5)) under the maximum entropy condition (Equation (4)) yields Equation (7), i.e., the partial derivative of the determinant is set to zero (Equation (8)).

(7) $$\frac{\delta \left\{\frac{M+1}{2}\ln\left[2\pi e+\frac{1}{2}\ln\left(\det R_{M+1}\right)\right]\right\}}{\partial_{R_{M+1}}}=0$$

(8) $$\frac{\partial \left(\det R_{M+1}\right)}{\partial R_{M+1}}=0$$

From Equation (8), it follows that the necessary and sufficient condition for Equation (9) to hold is that the last row of the left-hand matrix is a linear combination of the preceding rows.

(9) $$\det\left[\begin{array}{cccc} r_{0} & r_{1} & \ldots & r_{M-1}\\ r_{1} & r_{0} & \ldots & r_{M-2}\\ \vdots & \vdots & \ddots & \vdots \\ r_{M-1} & r_{M-2} & \ldots & r_{0} \end{array}\right]=0\overset{\mathrm{Then}}{\to }\left[\begin{array}{cccc} r_{0} & r_{1} & \ldots & r_{M-1}\\ r_{1} & r_{0} & \ldots & r_{M-2}\\ \vdots & \vdots & \ddots & \vdots \\ r_{M-1} & r_{M-2} & \ldots & r_{0} \end{array}\right]\left[\begin{array}{c} \theta_{1}\\ \theta_{2}\\ \vdots \\ \theta_{M} \end{array}\right]=0$$

The right-hand side of Equation (9) holds if and only if the following two conditions are simultaneously satisfied:

(10) $$\left[\begin{array}{cccc} r_{0} & r_{1} & \ldots & r_{M-1}\\ r_{1} & r_{0} & \ldots & r_{M-2}\\ \vdots & \vdots & \ddots & \vdots \\ r_{M-1} & r_{M-2} & \ldots & r_{0} \end{array}\right]\left[\begin{array}{c} \theta_{1}\\ \theta_{2}\\ \vdots \\ \theta_{M} \end{array}\right]=\left[\begin{array}{c} r_{1}\\ r_{2}\\ \vdots \\ r_{M} \end{array}\right]$$

(11) $$r_{M+1=}=\theta_{1}r_{M+}\theta_{2}r_{M-1}+\cdots +\theta_{M}r_{1}$$

Thus, once the first *M* parameter values are known, Equation (10) can be recursively applied to obtain Equation (11). By extrapolating the autocorrelation function according to the maximum entropy criterion, the effective data length is extended. Since data length is a key factor influencing the accuracy of spectral estimation, the power spectra obtained by the maximum entropy method are more accurate and exhibit higher resolution than those obtained by conventional approaches.

### 3.2. INPEFA Principle

**Figure 3:**
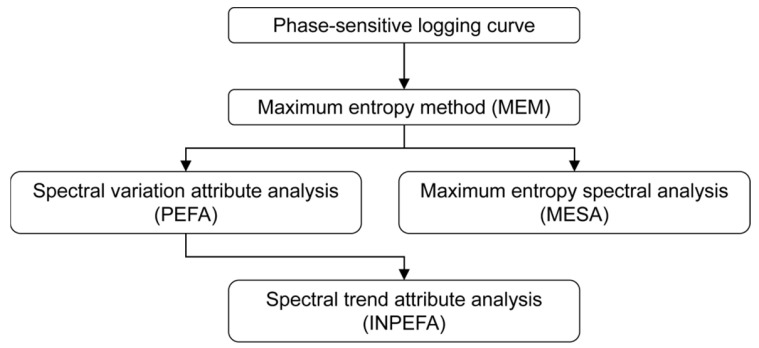
Flowchart of well log spectral analysis methods.

During Maximum Entropy Spectral Analysis, the autocorrelation function is extrapolated according to the maximum entropy criterion and compared with the actual logging data. This process yields the Prediction Error Filter Analysis (PEFA) curve, the integration of which produces the INPEFA curve [30]. The workflow of spectral analysis for logging curves is illustrated in Figure 3.

The key features of an INPEFA curve include the overall trend and the intermediate inflection points. In general, a positive trend (values increasing from left to right, with the curve rising) usually reflects a transgressive process under increasingly humid climatic conditions, whereas a negative trend (values decreasing from left to right, with the curve falling) corresponds to a regressive process under increasingly arid conditions. Inflection points in the curve indicate sequence boundaries or characteristic interfaces within sequences. Specifically, a negative inflection point (where the curve changes from rising to falling, corresponding to a negative peak in the PEFA curve) typically represents a flooding surface, while a positive inflection point (where the curve changes from falling to rising, corresponding to a positive peak in the PEFA curve) generally represents a sequence boundary [31,32].

### 3.3. Singular Spectrum Analysis

This study employed Singular Spectrum Analysis (SSA) [33,34], which extracts the trend, fluctuation, and noise components of the INPEFA curves through four steps: constructing the trajectory matrix, decomposing the trajectory matrix, grouping singular values, and reconstructing the time series.

1. Construction of the trajectory matrix

For a sequence of length *N*, an embedding window of length *M* and dimension *L* (*L* = *N* − *M* + 1) is defined to construct an *M* × *L* trajectory matrix *X*, which is calculated as follows:

(12) $$\begin{array}{c} X=[X_{1},X_{2},\cdots X_{L}]={\left(x_{ij}\right)}_{i,j=1}^{L,K}\;i=1,2,\cdots ,L;\;j=1,2,\cdots ,M;\;Xi={\left(x_{i},\cdots ,x_{i+M-1}\right)}^{T}\\ X=\left[\begin{array}{cccc} x_{1} & x_{2} & \ldots & x_{N-M+1}\\ x_{2} & x_{3} & \ldots & x_{N-M+2}\\ \vdots & \vdots & \ddots & \vdots \\ x_{M} & x_{M+1} & \ldots & x_{N} \end{array}\right] \end{array}$$

2. Decomposing the trajectory matrix

The trajectory matrix is decomposed by performing singular value decomposition (SVD) through eigenvalue analysis:

(13) $$X=U\overset{\Sigma }{\overset{︷}{\left[\begin{array}{cccccc} \sigma_{1} & 0 & \ldots & 0 & 0 & 0\\ 0 & \sigma_{2} & \ldots & 0 & 0 & 0\\ \vdots & \vdots & \ddots & 0 & 0 & 0\\ 0 & 0 & \ldots & \sigma_{M} & 0 & 0 \end{array}\right]}}V^{T}$$

where *U* and *V* are orthogonal matrices, and *Σ* is a diagonal matrix containing singular values *σ*_1_ ≥ *σ*_2_ ≥ ... ≥ *σ_L_* ≥ 0. Each singular value *σ_i_* corresponds to a component of the time series.

3. Grouping singular values

Singular values are sorted and grouped based on the singular entropy increment (Equation (14)) and the singular value contribution rate (Equation (15)). If the entropy increment Δ*E_i_* (Equation (14)) becomes stable after the *r*-th order, the first *r* singular values contain the main information. At this point, the cumulative contribution ratio *C_j_* (Equation (15)) further helps to distinguish trend, periodic, and noise components.

(14) $$∆E_{i}=-\left(\sigma_{i}/\sum_{k=1}^{M}\sigma_{k}\right)\log\left(\sigma_{i}/\sum_{k=1}^{M}\sigma_{k}\right)$$

(15) $$C_{j}=\sum_{r=1}^{j}\sigma_{r}/\sum_{r=1}^{M}\sigma_{r}\left(0\leq j\leq M\right)$$

4. Reconstructing the time series

Each group of singular values is transformed back into the time domain using the averaging diagonalization formula (Equation (16)), yielding the reconstructed time series *Zk* = {*Z*_1_, *Z*_2_, ..., *Z_M_*}. The original series is the sum of all reconstructed components.

(16) $$Z_{K}=\left\{\begin{array}{l} \frac{1}{k}\sum_{p=1}^{k}x_{p,k-p+1}\left(1\leq k<\min\left\{M,L\right\}\right)\\ \frac{1}{\min\left\{M,L\right\}}\sum_{p=1}^{\min\left\{M,L\right\}}x_{p,k-p+1}\left(\min\left\{M,L\right\}\leq k<\max\left\{M,L\right\}\right)\\ \frac{1}{N-k+1}\sum_{P=k-\max\left\{M,L\right\}+1}^{N-\max\left\{M,L\right\}+1}x_{p,k-P+1}\left(\max\left\{M,L\right\}\leq k<N\right) \end{array}\right.$$

## 4. Results

This study applied SSA in two stages to well log interpretation for the first time, aiming to improve the stability and geological interpretability of INPEFA curves. Based on GR data from 28 boreholes, with borehole 2007-1 as the main case (and 2007-4 as a supplementary example), we systematically illustrated the preprocessing of GR logs, first-stage SSA, MESA, and the construction of INPEFA curves, as well as the second-stage SSA processing of INPEFA curves and corresponding results.

### 4.1. SSA Preprocessing of GR Logs

**Figure 4:**
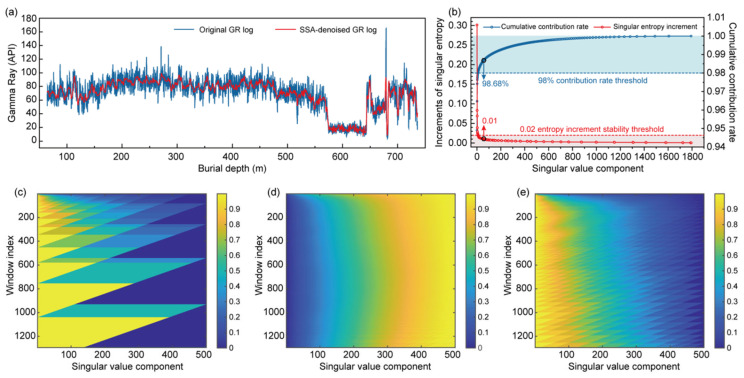
SSA preprocessing results of the GR logging curve from borehole 2007-1: (**a**) Comparison between the original and SSA-denoised GR curves; (**b**) cumulative contribution ratio and singular entropy increment curves; (**c**–**e**) Normalized sliding-window heatmaps showing the variation patterns of (**c**) singular entropy increments, (**d**) cumulative contribution ratio, and (**e**) contribution ratio for the first 500 singular values.

Raw GR logs are often affected by high-frequency noise and local outliers, which compromise the accuracy of subsequent INPEFA. To enhance data quality, SSA was applied to the GR data from all boreholes to extract the main components and suppress noise. Taking borehole 2007-1 as an example, SSA processing significantly reduces high-frequency fluctuations and improves the signal-to-noise ratio (Figure 4a).

During SSA-based denoising, the cumulative contribution ratio (CCR) rapidly increased with the addition of singular components, while the incremental singular entropy (ISE) quickly stabilized. For the first 56 singular values, the CCR reaches 98.68% and the ISE is 0.01, both within the preset thresholds (Figure 4b). This indicates that the majority of the signal information is captured by the first 56 singular values, with subsequent components mainly representing noise. Normalized sliding-window heatmaps of ISE, CCR, and contribution ratios (Figure 4c–e) further highlight the distinction between signal and noise. The rapid transition from yellow to blue in the normalized datasets indicates a sharp decay and stabilization of contribution with increasing singular value order (Figure 4c,e). The smooth gradient observed in the CCR heatmap demonstrates that the first singular value alone explains 94% of the variance, while the remaining singular values contribute little to the original sequence, confirming the necessity of removing high-frequency noise to improve data quality (Figure 4d).

**Figure 5:**
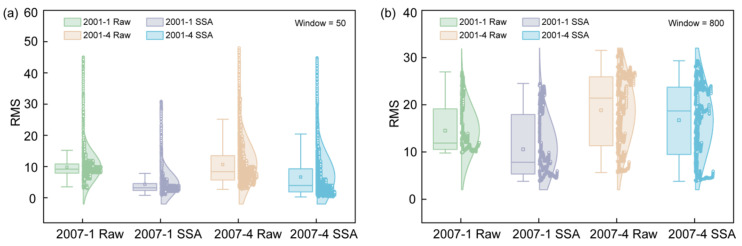
Boxplot statistics of RMS distributions for the raw and SSA-denoised GR curves from boreholes 2007-1 and 2007-4, with sliding window sizes of (**a**) 50 and (**b**) 800.

To comprehensively evaluate the performance of SSA denoising, root mean square error (RMS) and semi-violin–box plots were calculated for the raw and SSA-denoised GR logs using sliding windows of 50 and 800 (Figure 5).

At the small scale (window = 50), the RMS mean and interquartile range of the denoised logs are significantly reduced, and extreme outliers decrease (Figure 5a), indicating effective suppression of noise components. At the large scale (window = 800), the RMS differences between the raw and denoised logs are minor (Figure 5b), suggesting that SSA denoising primarily acts on local high-frequency noise while preserving long-term trends, thereby effectively retaining geological information.

In summary, SSA effectively separated the main and noise components of GR logs and provided a clear basis for determining the number of principal components as well as for analyzing the variations in component contributions within local windows.

### 4.2. MESA and INPEFA Results

**Figure 6:**
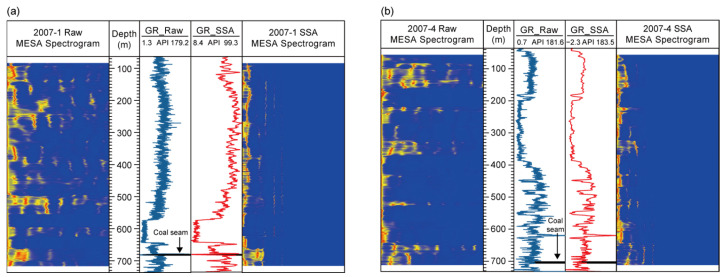
Comparison of MESA spectrograms and GR logs before and after SSA denoising for boreholes (**a**) 2007-1 and (**b**) 2007-4.

After preprocessing the GR logs, MESA (Section 3.1) was applied to both the raw and SSA-denoised GR data, which were depth-aligned before spectral analysis. The corresponding spectrograms were then generated (Figure 6).

The MESA spectrograms intuitively reveal the dominant sedimentary cycles at different depth intervals. In the SSA-denoised results, high-frequency noise is significantly reduced, and the dominant cycles become more distinct. For example, in borehole 2007-4 (Figure 6b), the Jurassic interval (0–478 m) is characterized by a continuous distribution of long-wavelength, high-energy bands, corresponding to low-amplitude and stable GR responses. With increasing depth, high-energy bands in the spectrogram gradually extend from the long- to short-wavelength zones, and the dominant cycles display multi-scale superposition, consistent with enhanced fluctuations in the GR logs. Within the coal-bearing interval (701–704 m), multiple energy peaks appear in the transition zone of the dominant frequency bands, reflecting frequent environmental changes and enhanced rhythmicity during coal formation.

**Figure 7:**
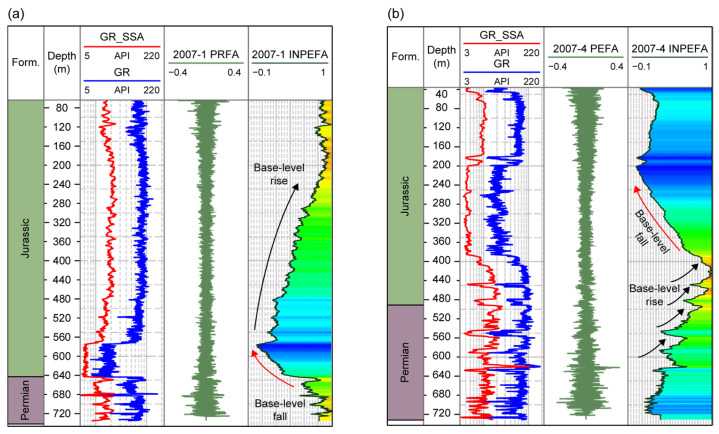
INPEFA results for boreholes (**a**) 2007-1 and (**b**) 2007-4. Red arrows indicate negative trends (base-level fall) in the INPEFA curve, while black arrows indicate positive trends (base-level rise).

Overall, the MESA spectrograms indicate a stratigraphic transition from the thick, stable Jurassic deposits to the multi-cyclic sedimentation of the Permian. This provides a foundation for identifying cycle-related trends (e.g., sea-level fluctuations) in INPEFA, for selecting window parameters during SSA decomposition of INPEFA curves, and for constructing the GR, INPEFA, and SSA_INPEFA curve systems used to extract coal-free zone logging responses. Based on the above MESA results, the extrapolated autocorrelation function was compared with the actual logging data to obtain the PEFA curve, whose integration yielded the INPEFA curve (Figure 7).

The INPEFA curves from boreholes 2007-1 and 2007-4 clearly reflect the periodic fluctuations of sea-level rise and fall (Figure 7). In the Jurassic interval, the curves exhibit fewer inflection points and smoother trends, whereas in the underlying Permian strata, inflection points increase markedly, reflecting frequent base-level changes (Figure 7a,b). The dense high-frequency inflection points in the Permian INPEFA curves reveal the orderly evolution of fluvial deposits within the fluvial-dominated delta plain subfacies, providing a clear basis for identifying the responses of coal-bearing depositional environments in logging data. However, despite the clarity of overall trends, the INPEFA curves still display dense local sawtooth fluctuations. To further highlight their stratigraphic responses, it is necessary to perform a second-stage SSA (SSA-2) to extract more geologically meaningful cyclic features.

### 4.3. SSA Processing of INPEFA Curves

**Figure 8:**
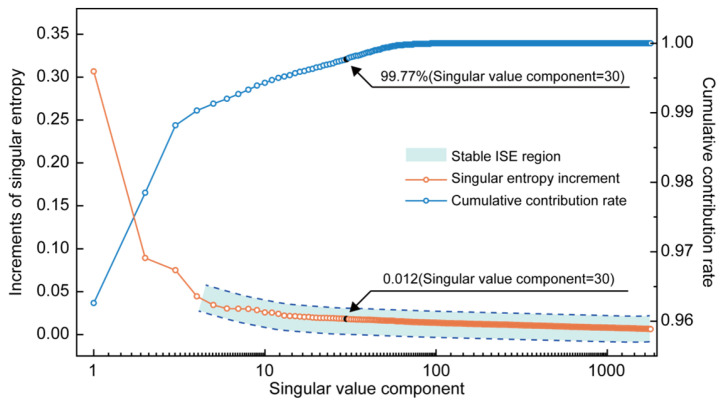
Singular value contribution ratio curve of INPEFA SSA decomposition.

Based on the principles of SSA, a second-stage decomposition was applied to the INPEFA curves to extract their trend and principal components, with borehole 2007-1 as an example. During the construction of the trajectory matrix, the window length *M* was set to 1793 according to the data length and the dominant cycles identified by MESA. Subsequently, the trajectory matrix was decomposed using SVD, and the singular value contribution curve was plotted (Figure 8).

**Figure 9:**
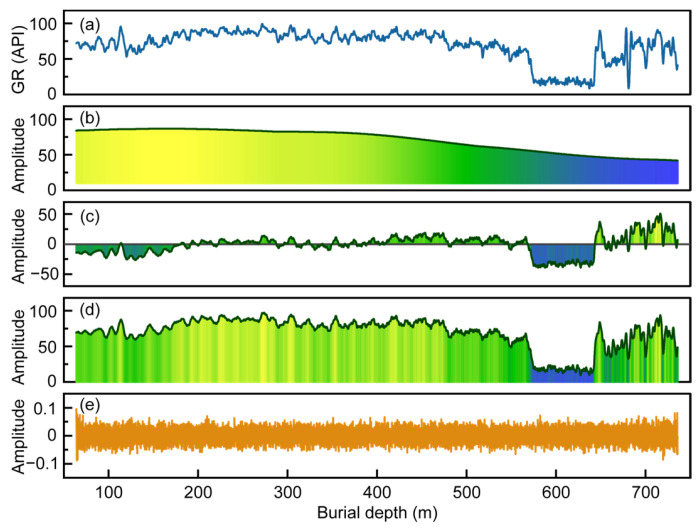
SSA decomposition results of the INPEFA curve from borehole 2007-1: (**a**) GR logging curve; (**b**) trend component reconstructed using the first singular value; (**c**) fluctuation component reconstructed using the 2nd to 30th singular values; (**d**) superposition of the trend and fluctuation components; (**e**) noise component reconstructed from the higher-order singular values.

The contribution of the first singular value is 96.26%, and the incremental singular entropy stabilizes after the 30th order, with the cumulative contribution reaching 99.7% (Figure 8). Accordingly, the first singular value was used to reconstruct the trend component, the 2nd to 30th singular values were used to reconstruct the fluctuation component, and the remaining higher-order singular values were used to extract noise. Each component was restored to a one-dimensional depth sequence through Equation (16), resulting in the trend, fluctuation, and noise curves (Figure 9).

Thus, the trend, fluctuation, and noise components of the INPEFA curve for borehole 2007-1 were successfully extracted. By extending this procedure to all 28 boreholes in the study area, the minimum singular value order *N* required to denoise each INPEFA curve was obtained (Table 2). This parameter provides a quantitative measure that, to some extent, reflects the depositional complexity of the stratigraphic intervals encountered in different boreholes.

## 5. Discussion

### 5.1. Engineering Potential of the Minimum Singular Value Order

The stability of depositional environments directly influences the structural complexity of strata. Traditional evaluations of complexity mainly rely on parameters such as the number of interbeds and single-bed thickness interpreted from well logs [35,36,37]. However, these methods are constrained by the accuracy of lithological interpretation and cannot fully capture the information contained in logging curves. Therefore, this study proposes the minimum singular value order (*N*), obtained from denoising INPEFA curves through second-stage SSA, as a potential auxiliary quantitative indicator of depositional complexity. It should be noted that *N* is a parameter that naturally arises during the second SSA decomposition of the INPEFA curve, rather than an independently constructed evaluation system, and its role is mainly to reflect the relative complexity of the depositional sequence.

#### 5.1.1. Theoretical Basis and Statistical Results

In Singular Spectrum Analysis, each singular value and its associated eigenvector represent a dominant variation mode of the curve. If the curve structure is simple and highly regular, only a few singular values are sufficient for effective reconstruction. This indicates that the curve is governed by a small number of controlling depositional factors, corresponding to stable environments and well-organized stratigraphic stacking. Conversely, if lithological variations occur frequently, more singular values are required for reconstruction to capture the full variability, suggesting that the curve is influenced by multiple controlling modes and reflecting complex and unstable depositional environments. Thus, the minimum singular value order *N* can be regarded as a quantitative measure of stratigraphic complexity: a small *N* suggests strong regularity and depositional stability, whereas a large *N* implies frequent lithological variations and structural complexity.

**Figure 10:**
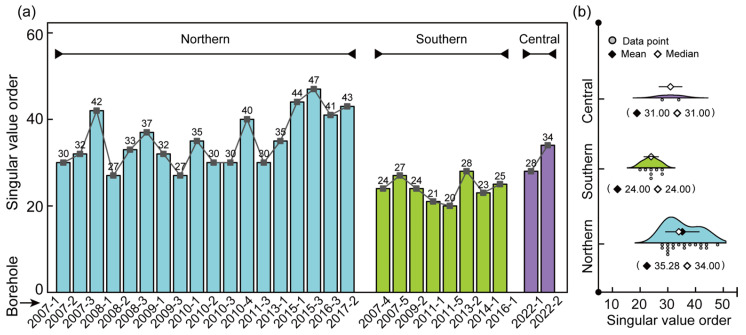
(**a**,**b**) Alluvial diagram of principal component (singular value) distribution by borehole and subregion in the study area.

The study area was divided into a northern coal-bearing zone, a central coal-free zone, and a southern coal-bearing zone, and the minimum singular value order *N* required for INPEFA curve denoising in each borehole was statistically analyzed (Figure 10; Table 3). The results show that in the northern coal-bearing zone, the 18 boreholes exhibit *N* values ranging from 27 to 47, with a mean of 35.28 and a median of 34, and the distribution is relatively scattered, indicating more complex stratigraphic structures and frequent lithological changes. In the southern coal-bearing zone, *N* values range from 20 to 28, with a mean of 24 and a median of 24, showing a concentrated distribution that reflects stable depositional rhythms and more continuous strata. In the central coal-free zone, the two boreholes both yield an *N* value of 31. Although the sample size is limited, the results suggest that the stratigraphic complexity in this area lies between that of the northern and southern parts. Overall, the southern strata are more complete with better single-bed continuity, whereas the northern strata frequently display missing intervals and more complex structures.

#### 5.1.2. Geological and Engineering Implications

**Figure 11:**
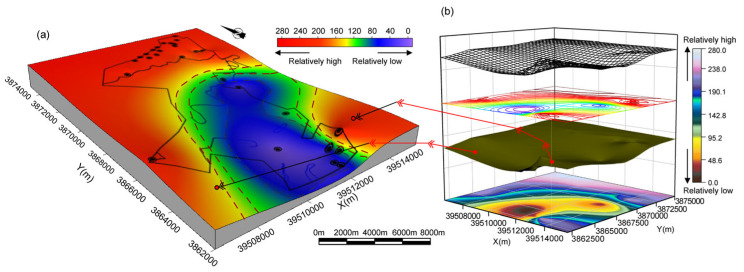
(**a**) Boreholes located on paleotopographic highs generally show larger minimum singular value orders (*N*). (**b**) Approximate post-depositional paleogeomorphology reconstructed from the residual thickness of the Shihezi Formation.

To further illustrate the stratigraphic complexity reflected by the minimum singular value order (*N*), the post-depositional paleogeomorphology reconstructed approximately from the residual thickness of the Lower Shihezi Formation was used to visualize the relationship between *N* and stratigraphic integrity (Figure 11b).

Based on the statistical results shown in Figure 10 and Table 3, boreholes with larger *N* values are mainly concentrated in the northern part of the mining area, spatially corresponding to the high paleotopographic zones (Figure 11a). These areas experienced stronger uplift and erosion during the post-depositional stage, which disrupted the original stratigraphic stacking relationships and caused the INPEFA curves to exhibit more frequent fluctuations; consequently, more principal components were required during SSA reconstruction. Meanwhile, boreholes with high *N* values display superimposed multi-wavelength high-energy bands in the MESA spectrograms (Figure 6a,b) and dense high-frequency inflection points on the INPEFA curves (Figure 7a,b), further indicating more frequent lithological variations. In contrast, boreholes with lower *N* values are mostly located in paleotopographic lows (southern part of the mining area, Figure 11a). These locations provided greater accommodation space for sediment accumulation, resulting in more completely preserved sedimentary sequences and reflecting a stable depositional environment with stronger stratigraphic regularity.

Thus, the minimum singular value order (*N*) essentially reflects the minimum number of signal components required to separate the noise term in the INPEFA curve, serving as an indirect, mathematically derived quantitative indicator of stratigraphic complexity and the frequency of lithological variations. Boreholes with larger *N* values generally correspond to strata that experienced stronger post-depositional disturbances, more frequent lithological changes, and more pronounced permeability contrasts, which together indicate an overburden framework with a higher potential for mine water inrush under mining-induced stress. In contrast, areas with smaller *N* values typically correspond to more stable depositional environments and more regularly organized sedimentary sequences, whose lithological architecture often consists of stable and relatively continuous mudstone–sandstone interbeds. Such a continuous and coherent overburden structure tends to fail primarily under mining-induced stress, corresponding to a lower-risk hydrogeological setting. Therefore, compared with traditional complexity evaluations based on core statistics [38], the *N* value is obtained directly from the mathematical decomposition of the logging curve itself, thereby reducing uncertainties associated with subjective lithological interpretation.

In summary, the minimum singular value order *N*, obtained from SSA decomposition of INPEFA curves, provides a useful proxy for stratigraphic complexity and lithological variation frequency across different regions. This indicator offers a new perspective for the quantitative evaluation of mine water inrush hazards and roof aquifer richness in coal mines. However, it should be emphasized that *N* mainly reflects stratigraphic structure and lithological variability, and its direct applicability to the genesis of coal-free zones remains limited. In future work, *N* can be integrated with other geological and logging parameters to further improve the evaluation of water inrush hazards and related engineering challenges during coal mining in coal-free zones and adjacent areas.

### 5.2. Genesis Analysis and Detection of Coal-Free Zones

In Section 4.3, SSA decomposition of INPEFA curves extracted the trend, fluctuation, and noise components from 28 boreholes (Figure 9b,c). By superimposing the trend and fluctuation components, a Trend–Fluctuation Composite (TFC) curve was constructed (Figure 9d). This curve eliminates sawtooth noise, exhibits improved cyclic continuity, and enhances the identification and tracking of depositional rhythms.

#### 5.2.1. Genesis of Coal-Free Zones

**Figure 12:**
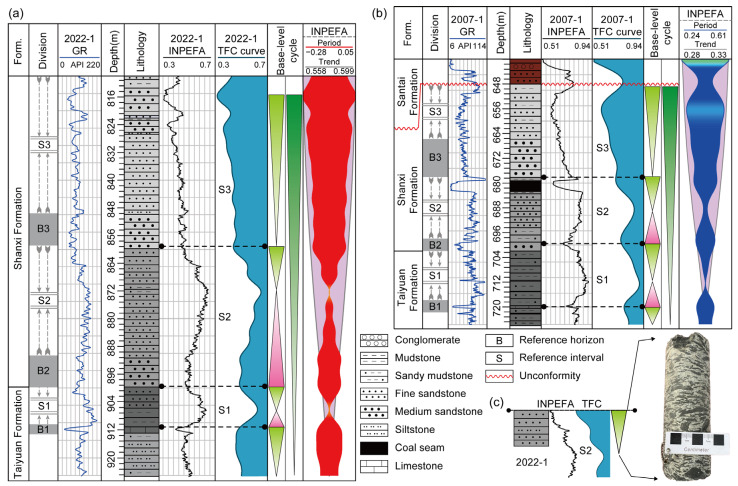
Multi-curve framework illustrating coal-free zone genesis: (**a**) INPEFA and TFC curves for borehole 2022-1 in the coal-free zone; (**b**) Comparison of INPEFA and TFC curves for borehole 2007-1 in the coal-bearing zone; (**c**) Core sample from the S2 interval showing mudstone clasts and muddy conglomerate.

Based on SSA–INPEFA, this study established a five-curve recognition framework consisting of GR, INPEFA, TFC, INPEFA_trend, and INPEFA_period to characterize the logging responses of coal-free zones. Borehole 2022-1 (coal-free) and borehole 2007-1 (coal-bearing) were selected for comparison, with all curves aligned in depth and integrated with core data to analyze the genesis of coal-free zones (Figure 12).

Taking the third limestone (B1) at the top of the Taiyuan Formation and the two overlying medium sandstones of the Shanxi Formation (B2 and B3) as stratigraphic markers, the sequence was divided into three intervals: S1, S2, and S3. In the S1 interval (B1–B2), both the TFC and INPEFA curves display complete upward and downward half-cycles, with B1 and B2 corresponding to positive inflection points. Their strong regional correlation indicates that B1 can serve as the basal boundary for analyzing the genesis of coal-free zones, while B2 and B3 can be used as subdivision markers.

In the S2 interval (B2–B3), comparison of boreholes 2007-1 and 2022-1 reveals significant differences in INPEFA and TFC curves (Figure 12). Although both boreholes exhibit complete cyclicity, the coal-related low-GR anomalies strongly affect curve morphology. In borehole 2007-1, the INPEFA curve shows obvious discontinuous transitions at coal seam inflection points, while the TFC curve displays balanced upward and downward half-cycles, with smooth overall trends and fewer inflection points, reflecting relatively stable depositional evolution in the S2 interval (Figure 12b). By contrast, in borehole 2022-1, the INPEFA curve exhibits better half-cycle continuity at negative inflection points, and the TFC curve shows shorter downward half-cycles, dense inflection points, and more frequent fluctuations, indicating more active environmental changes in coal-free intervals (Figure 12a). In addition, core samples from borehole 2022-1 reveal mud conglomerates containing mudstone rip-up clasts in the downward half-cycle of the S2 interval (Figure 12c), further indicating that contemporaneous peat deposits were scoured and transported by fluvial processes before consolidation, preventing coal preservation.

Taken together, the combined evidence from logs and cores demonstrates that the genesis of coal-free zones was mainly controlled by regional base-level changes. Enhanced fluvial dynamics introduced abundant clastic material, which terminated the development of peat swamps [39] and scoured peat layers in main channels [40], ultimately forming coal-free zones.

In summary, comparative analysis of INPEFA and TFC curves reveals a set of diagnostic logging features for coal-free zones: INPEFA curves with dense inflection points and strong half-cycle continuity; TFC curves with marked differences between upward and downward half-cycle lengths, frequent inflections, and stronger fluctuations; and GR logs lacking low-GR anomalies. These features, consistent with core evidence of scoured peat layers and inhibited coal preservation, confirm that the SSA–INPEFA-based multi-curve recognition framework not only elucidates the genesis of coal-free zones but also provides a reliable basis for their logging response characterization and practical identification.

#### 5.2.2. Fine-Scale Detection of Coal-Free Zones

The coal-free zone was formed by fluvial scouring, where the depositional style is characterized by sand-rich and mud-poor sediments, while coal-bearing areas exhibit mud-rich and sand-poor assemblages. This lithological contrast directly leads to higher resistivity in the coal-free zone compared with the coal-bearing zone. Based on this principle, a mine-scale three-dimensional direct-current (3D DC) resistivity survey was conducted using the underground DC system along the track and belt roadways in the western wing of the Qiyi mining area, where the roadways cross the coal-free zone. The details of the geophysical survey are provided in Appendix A.

**Figure 13:**
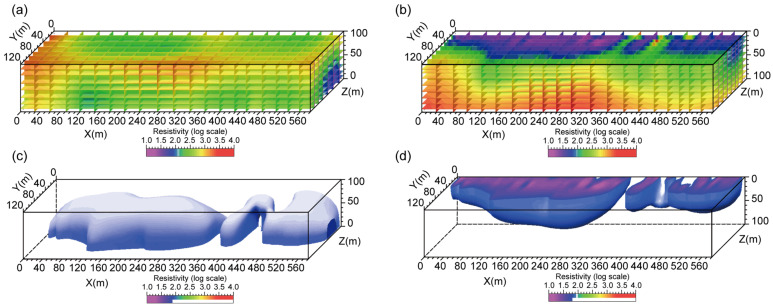
In-mine 3D DC resistivity data volumes: (**a**,**b**) Resistivity slices of the roof and floor showing clear resistivity contrasts across the coal-free boundary; (**c**,**d**) Isosurface distribution of low-resistivity zones indicating spatial variations in lithology.

Integrated analysis of the 3D resistivity slices of the roof (Figure 13a,c) and floor (Figure 13b,d) shows a clear resistivity contrast at the coal-free boundary: low resistivity on the coal-bearing side and high resistivity on the coal-free side, with a sharply defined transition. The resistivity variation reflects the underlying lithological changes. The coal-bearing area contains higher mud content and lower sand content, resulting in generally lower resistivity; in contrast, the coal-free area contains less mud and more sand, leading to higher resistivity. This lithological difference corresponds to the lateral migration and scouring processes of paleo-channels, and the coal-free boundary essentially represents the paleo-channel scour margin. Furthermore, the resistivity signature along this boundary can be compared with the S2 interval logging responses of the coal-free borehole 2022-1 (Figure 12a) and the coal-bearing borehole 2007-1 (Figure 12b), as well as their corresponding core characteristics (Figure 12c).

Combining the 3D DC resistivity results with logging and core data, the S2 interval in coal-free borehole 2022-1 shows no low GR anomaly, and the INPEFA and TFC curves exhibit dense inflection points and enhanced half-cycle fluctuations. The core contains abundant mudstone rip-up clasts, all of which indicate that the peat-swamp environment experienced strong hydrodynamic disturbance, unfavorable for peat accumulation and preservation (Figure 12a,c). Correspondingly, the resistivity slices display extensive high-resistivity zones (Figure 13a,b), suggesting increased sand content and decreased mud content, consistent with the sandy lithofacies revealed by the core. In contrast, the S2 interval in the coal-bearing borehole 2007-1 shows distinct low GR and INPEFA anomalies due to the presence of the coal seam; the TFC curve exhibits nearly symmetrical half-cycles with fewer inflection points, indicating a relatively stable depositional environment (Figure 12b). The corresponding resistivity slices show continuous low-resistivity bands (Figure 13c,d), representing a mud-rich facies and a stable peat-swamp environment during the late stage of the depositional cycle. This comparison demonstrates that the high/low resistivity distribution agrees well with the logging-response differences between coal-bearing and coal-free zones.

In summary, the integration of well logs, core data, and 3D resistivity surveys, together with the SSA–INPEFA multi-curve recognition system and resistivity slices, demonstrates that coal-free zones were mainly controlled by regional base-level changes. Paleochannel scouring prevented the preservation of peat layers and led to the development of coal-free zones. On this basis, resistivity contrasts across coal-free boundaries enabled fine-scale delineation of coal-free zones. These findings provide a practical basis for the rational layout of working faces in new mining areas and for the prevention of water hazards associated with coal-free zones.

### 5.3. Summary

This study, motivated by the engineering challenge of resource depletion in the No. 3 coal seam of the Tianchen Coal Mine and the constraints imposed by the extensive coal-free zone on the layout of new working faces, systematically investigates the genesis of the coal-free zone and its fine-scale delineation. A two-stage SSA was applied for the first time to logging data to achieve noise reduction and principal component extraction. Combined with MESA, the dominant frequencies and sedimentary cycles recorded in the logging curves were identified, thereby enhancing their geological interpretability. Building on this, a TFC was innovatively constructed by superimposing the trend and fluctuation components of the INPEFA curve, which significantly improves the expression of sedimentary rhythms and inflection-point characteristics.

In addition, this study introduces the minimum singular value order (*N*), derived from the second-stage SSA decomposition of the INPEFA curve, as a quantitative indicator reflecting stratigraphic structural complexity and the frequency of lithological variations across different regions. It should be noted that *N* primarily captures the complexity and variability of stratigraphic structures and has limited direct diagnostic capability for interpreting the genesis of coal-free zones. Nonetheless, as a parameter naturally arising from the logging-curve analysis workflow, *N* offers new perspectives for the quantitative evaluation of mine water inrush hazards and roof aquifer richness. Future work may integrate N with additional geological and logging parameters to further improve assessments of water inrush risk and related engineering challenges in coal-free zones and adjacent mining areas.

Based on the aforementioned methods and indicators, and combined with a multi-curve integrated identification framework, the principal logging responses of the coal-free zone were systematically characterized. Comparative analysis with core data confirms that the coal-free zone primarily results from regional base-level fluctuations and scour by paleo-channels, which inhibited the preservation of peat and coal seams. Furthermore, by incorporating multiple geophysical datasets—including 3D DC resistivity—the boundary of the coal-free zone was delineated with high precision. These findings not only enhance the theoretical understanding of coal-free zone genesis and its logging-response characteristics but also provide a basis for the quantitative evaluation of mine water inrush hazards and roof aquifer hydrogeological conditions.

## 6. Conclusions

1. This study addressed the practical problem of resource depletion in the No. 3 coal seam of the Tianchen Coal Mine and the restriction posed by extensive coal-free zones on the layout of new working faces. A multi-curve recognition framework was developed, and by integrating core data with 3D DC resistivity surveys, the genetic mechanisms of coal-free zones were clarified and their boundaries delineated with high precision.
2. The results indicate that coal-free zones are characterized by dense inflection points and frequent cyclic fluctuations in TFC curves and by the absence of obvious low values in GR curves, distinguishing them from the relatively stable characteristics of coal-bearing zones. The formation of coal-free zones was mainly due to inhibited peat-swamp development, followed by fluvial scouring and reworking of peat deposits, which prevented coal preservation and ultimately resulted in coal-free belts.
3. For the first time, this study systematically introduced two-stage SSA into well log processing. Combined with MESA, this significantly enhanced the identification of sedimentary cyclicity and dominant frequencies. On this basis, a TFC curve was innovatively constructed, enhancing the capacity of logs to capture depositional rhythms and inflection features. In addition, the minimum singular value order (*N*), obtained from SSA decomposition of INPEFA curves, effectively reflects stratigraphic complexity and the frequency of lithological variations across different regions. Although *N* has limited direct applicability for determining the genesis of coal-free zones, it offers a new controlling factor for related engineering geological problems and provides practical guidance for coal-free zone detection and water hazard prevention in similar mining areas.

## Figures and Tables

**Table 1:** Borehole location parameters in the study area.

No.	Borehole Name	X	Y	No.	Borehole Name	X	Y
1	2007-1	39,512,294.12	3,873,802.30	15	2010-4	39,510,565.07	3,872,949.67
2	2007-2	39,511,949.17	3,873,620.44	16	2011-1	39,512,111.00	3,862,529.55
3	2007-3	39,512,144.41	3,873,360.68	17	2011-3	39,513,608.97	3,874,103.27
4	2007-4	39,512,501.59	3,862,949.15	18	2011-5	39,512,880.23	3,863,344.15
5	2007-5	39,513,007.90	3,865,963.20	19	2013-1	39,513,091.67	3,873,509.96
6	2008-1	39,511,753.86	3,873,016.88	20	2013-2	39,512,501.43	3,863,394.01
7	2008-2	39,511,417.38	3,873,347.66	21	2014-1	39,506,795.13	3,865,504.56
8	2008-3	39,513,123.55	3,873,902.64	22	2015-1	39,509,606.83	3,871,932.92
9	2009-1	39,511,270.18	3,872,883.77	23	2015-3	39,512,838.67	3,872,534.20
10	2009-2	39,512,046.62	3,862,753.99	24	2016-1	39,511,369.09	3,864,676.04
11	2009-3	39,512,851.56	3,874,108.54	25	2016-3	39,513,990.55	3,872,530.19
12	2010-1	39,513,869.69	3,874,635.89	26	2017-2	39,513,780.26	3,872,717.43
13	2010-2	39,513,223.12	3,873,722.54	27	2022-1	39,513,504.73	3,869,284.11
14	2010-3	39,510,991.56	3,873,186.22	28	2022-2	39,513,226.42	3,869,984.38

**Table 2:** Minimum singular value order *N* for INPEFA curve noise removal in each borehole.

No.	Borehole	*N*	No.	Borehole	*N*	No.	Borehole	*N*
1	2007-1	30	11	2009-3	30	21	2014-1	24
2	2007-2	32	12	2010-1	40	22	2015-1	21
3	2007-3	42	13	2010-2	30	23	2015-3	20
4	2007-4	27	14	2010-3	35	24	2016-1	28
5	2007-5	33	15	2010-4	44	25	2016-3	23
6	2008-1	37	16	2011-1	47	26	2017-2	25
7	2008-2	32	17	2011-3	41	27	2022-1	28
8	2008-3	27	18	2011-5	43	28	2022-2	34
9	2009-1	35	19	2013-1	24			
10	2009-2	30	20	2013-2	27			

**Table 3:** Descriptive statistics of singular value order (*N*) after noise removal for each borehole.

Location	*N*	Mean	Standard Deviation	Sum	Minimum	Median	Maximum
Northern	18.00	35.28	6.19	635.00	27.00	34.00	47.00
Southern	8.00	24.00	2.73	192.00	20.00	24.00	28.00
Central	2.00	31.00	4.24	62.00	28.00	31.00	34.00

**Figure A1:**
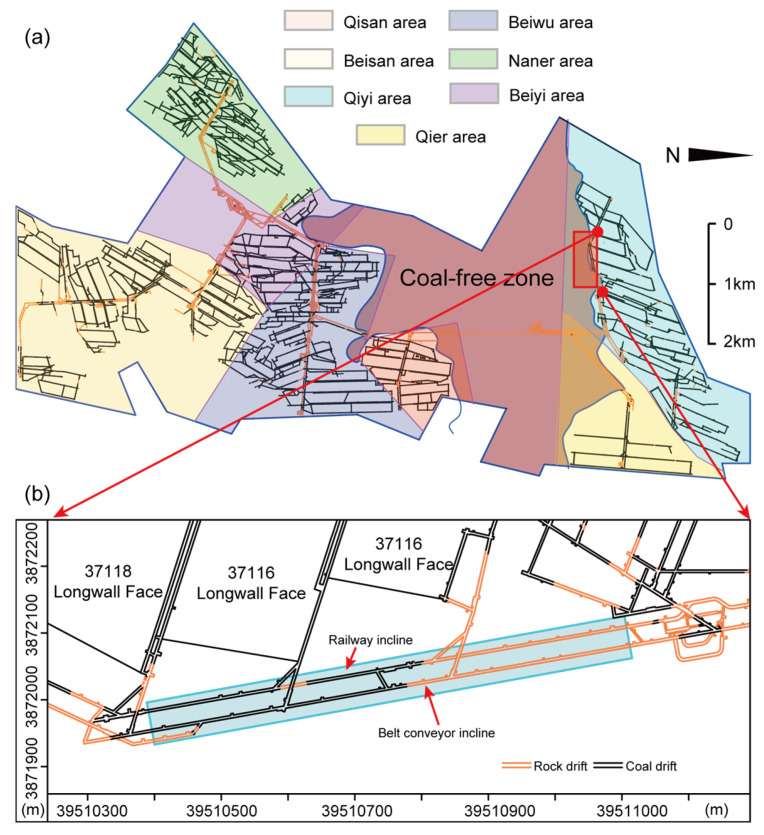
Location of the in-mine 3D DC resistivity survey in the Qiyi mining area: (**a**) Spatial extent of the coal-free zone and adjacent mining areas; (**b**) Survey area arranged along the railway incline and belt conveyor incline across the coal-free boundary.

**Figure A2:**
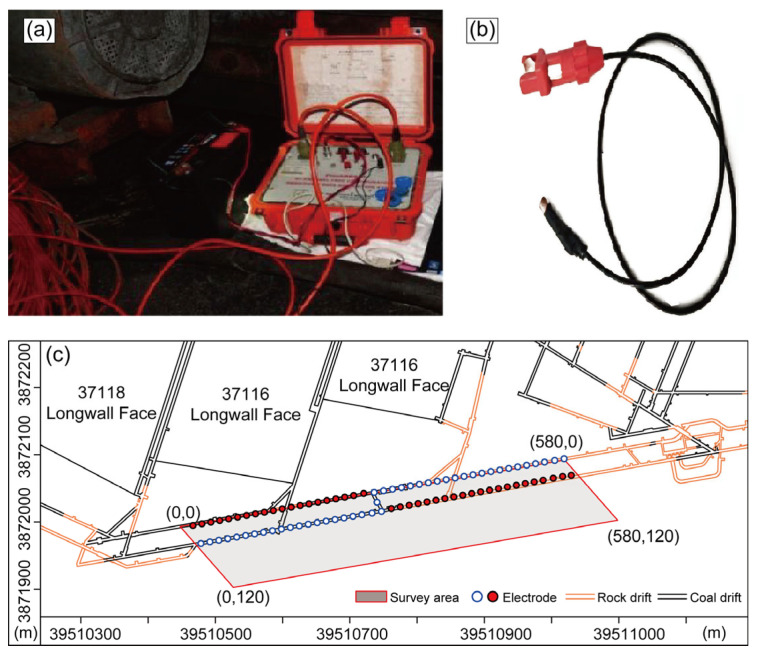
Instruments and electrode arrangement for the in-mine 3D DC resistivity survey: (**a**) WJDJ-3 resistivity meter (W.T.S Limited, Guangdong, China); (**b**) Electrode connection cables; (**c**) Schematic layout of the 10 m-spacing electrode array arranged along the railway incline and belt conveyor incline.

## Data Availability

The data that support the findings of this study are available upon reasonable request from the authors.

## Appendix A. Details of the In-Mine 3D DC Resistivity Survey

The 3D DC resistivity survey was conducted in the western wing of the Qiyi mining area (Figure A1a). The aim was to characterize the electrical structure of the No. 3 coal seam roof and floor and to delineate the boundary of the coal-free zone. The railway incline and belt conveyor incline passing through this zone (Figure A1b) provided favorable locations for underground instrument deployment. In addition, the survey area belongs to the Shanxi Formation of the Permian System (S2 interval in Figure 12a,b). If peat layers were eroded by paleo-channels during deposition, the sandstone–mudstone proportions would shift toward sand enrichment, producing notable resistivity variations. This provides the physical basis for geophysical identification of the coal-free boundary.

The survey employed the WJDJ-3 high-density DC resistivity system (Figure A2a). Electrodes and connecting cables (Figure A2b) were deployed along the railway incline and belt conveyor incline, covering a total length of 1190 m with 120 electrodes spaced at 10 m intervals (layout shown in Figure A2c). To ensure data quality, each measurement point was acquired at least twice. All power sources in adjacent roadways were switched off during data acquisition to minimize electromagnetic interference.

Data processing consisted of three sequential steps: quality control, inversion model construction, and 3D inversion. First, raw data were processed by removing outliers and applying mild smoothing to suppress random noise while preserving useful signals. Next, a 3D mesh model consistent with the survey layout was constructed based on the 10 m electrode spacing. Finally, a smoothness-constrained least-squares inversion was carried out. By adjusting the damping factor and the horizontal and vertical smoothing constraints, the mismatch between measured and forward-modeled data was progressively reduced, typically converging to a stable root-mean-square error after 3–5 iterations. The resulting 3D resistivity volumes of the roof and floor strata (Figure 13) provide a reliable basis for the refined delineation of the coal-free boundary.
